# Optimization of Ultrasound-Assisted Extraction of Phenolics from *Sideritis raeseri* Using Response Surface Methodology

**DOI:** 10.3390/molecules26133949

**Published:** 2021-06-28

**Authors:** Katarina Šavikin, Jelena Živković, Teodora Janković, Nada Ćujić-Nikolić, Gordana Zdunić, Nebojša Menković, Zorica Drinić

**Affiliations:** Institute for Medicinal Plant Research “Dr. Josif Pančić”, Tadeuša Košćuška 1, 11000 Belgrade, Serbia; ksavikin@mocbilja.rs (K.Š.); jzivkovic@mocbilja.rs (J.Ž.); tjankovic@mocbilja.rs (T.J.); ncujic@mocbilja.rs (N.Ć.-N.); gzdunic@mocbilja.rs (G.Z.); nmenkovic@mocbilja.rs (N.M.)

**Keywords:** *Sideritis*, extraction optimization, UAE, phenolics, hypolaetin and isoscutellarein derivatives

## Abstract

In this study we define the optimal conditions for ultrasound-assisted extraction of bioactive polyphenols from *S. raeseri* aerial parts using response surface methodology. The influence of ethanol concentration (10–90%), extraction temperature (20–80 °C), extraction time (10–60 min), and solid-to-solvent ratio (1:10–1:50) on total phenolic content as well as on content of individual flavonoids, and hypolaetin and isoscutellarein derivatives was studied. For the experimental design, a central composite design was chosen. In the obtained extracts, the following ranges of targeted compounds were detected: total phenol from 19.32 to 47.23 mg GAE/g dw, HYP from 1.05 to 11.46 mg/g dw, ISC 1 from 0.68 to 10.68 mg/g dw, and ISC 2 from 0.74 to 15.56 mg/g dw. The optimal extraction conditions were set as: ethanol concentration of 65%, extraction time of 50 min, extraction temperature of 63 °C, and solid-to-solvent ratio of 1:40. Contents of TP, HYP, ISC 1, and ISC 2 in optimal extracts were 47.11 mg GAE/g dw, 11.73 mg/g dw, 9.54 mg/g dw, and 15.40 mg/g dw, respectively. Experimentally set values were in good agreement with those predicted by the response surface methodology model, indicating suitability of the used model, as well as the success of response surface methodology in optimizing the conditions of the extraction.

## 1. Introduction

The genus *Sideritis* (Lamiaceae) comprises over 150 species, which are distributed throughout the Mediterranean region, the Balkan Peninsula, and the Middle East. Among them, *Sideritis raeseri* Boiss. and Heldr subsp. *Raeseri*—also known as mountain tea, shepherd’s tea, or ironwort—is widely used in folk medicine in those regions as a tea [1,2]. The tea is prepared from flowering aerial parts, and is mainly used to alleviate the symptoms of common colds, coughs, and bronchitis, as well as in the treatment of gastrointestinal disorders and inflammations, and as a tonic [3].

Diverse chemical composition of *Sideritis* species’ aerial parts has been reported, and includes phenolic acids, flavonoids, phenylpropanoids, iridoid glycosides, diterpenes, and essential oils [2,4,5,6].

Due to such diverse chemical composition, many biological activities are reported for *Sideritis* species, such as anti-inflammatory, spasmolytic, gastroprotective, antimicrobial, hypotensive, vasorelaxant, analgesic, antioxidant etc. [3,7,8,9]. Antioxidant, anti-inflammatory, and antimicrobial activities of the extracts are mainly connected with phenylpropanoids and flavonoids, while diterpenoids contribute to the antibacterial, cytotoxic, neuroprotective, and antitumor activities [3,7].

With the aim to obtain good yield, and high levels of bioactive compounds in the extract, it is necessary to optimize the extraction conditions [10]. Parameters such as type of solvent, type of extraction, extraction temperature and time, particle size, and solid-to-solvent ratio may influence the extract yield, as well as the amount of bioactive compounds. Usually, food-grade solvents such as water, ethanol, or their mixtures are used for the preparation of extracts. Ultrasound-assisted extraction, as a more efficient and time-saving technique compared to conventional techniques, has been widely applied for the extraction of bioactive compounds from different natural matrices [11]. In our study we have applied ultrasound-assisted extraction as a ”green“ or ”environmentally friendly“ technique for the extraction of phenolic compounds—known to be one group of compounds responsible for the biological activity of *S. raeseri*’s aerial parts.

According to data from the literature, extensive statistical studies dealing with the optimization of wild-growing *Sideritis* spp. extraction is missing. *Sideritis* spp. are traditionally used in several countries—such as Greece, Turkey, North Macedonia, and Albania—and *S. raeseri* is the most widely used species of the genus *Sideritis*. However, studies on optimizing the extraction of flavonoids from *S. raeseri*—which certain studies have shown significantly contribute to its biological activity—is lacking. Alipieva et al. [12] studied the influence of the extraction method on cultivated *Sideritis* spp. using methanol as a solvent, while Tsibranska et al. [13] tested the influence of the solvent, using maceration in fixed time and drug-to-solvent ratio. Both studies recorded total polyphenols or flavonoid content as final responses.

Considering the aforementioned, the main objective of this research was to apply ultrasound-assisted extraction in order to obtain *S. raeseri* extract with high content of phenolic compounds. In order to examine the impact of four parameters—i.e., extraction time and temperature, solid-to-solvent ratio, and ethanol concentration—on total polyphenol content, and on the content of individual bioactive compounds—i.e., hypolaetin and isoscutellarein derivatives known for their anti-inflammatory activity in extracts from *S. raeseri* aerial parts—the response surface methodology (RSM) was applied.

## 2. Results and Discussion

In order to estimate the optimization of the UAE process of *Sideritis raeseri*, we use the RSM as a compilation of mathematical and statistical analyses established on the fit of polynomial equations to the experimental data [14]; this describes well the behaviour of the dataset, aiming to make statistical previsions; since it reduces time, space, and raw material usage, it is more favourable than the traditionally used single parameter optimization [15]. Such a model of the optimization of the UAE process of *Sideritis* species is lacking. A comparison of extraction methods such as maceration, ultrasound-assisted extraction, and microwave-assisted extraction was conducted by Alipieva et al. [12], where the yield and the amount of total phenolics (TP) and total flavonoids (TF) were markers of the extraction quality. Taking into account that the maceration took 24 h and 50 mL/g solvent in their study, as opposed to 1 h and 15 mL/g solvent when they applied UAE, the authors pointed to UAE as the optimal procedure for the methanol extraction of *Sideritis* spp. Tsibranska et al. [13] studied the influence of the ethanol concentration on the extract yield and the TP and TF contents from cultivated hybrid *Sideritis scardica × Sideritis syriaca*, using maceration with fixed time and drug-to-solvent ratio. The maximum TP and TF content was obtained by the authors using a water–ethanol solvent ratio of 20:80.

To the best of our knowledge, this is the first paper dealing with the optimization of UAE based on the RSM, and calculating individual biologically active flavonoids from *Sideritis* spp. as dependent variables. Different extraction conditions promote greater extraction of different groups of compounds; thus, application of the optimal extraction procedure should achieve high recovery of target compounds, together with their minimal degradation [16]. In our study, we applied a central composite design (CCD), with four variables tested at five levels (Table 1), in order to obtain an extract of *S. raeseri* aerial parts with the highest content of 4′-*O*-methylhypolaetin-7-*O*-[6‴-*O*-acetyl-β-d-allopyranosyl (1→2)]-β-d-glucopyranoside (HYP), isoscutellarein 7-*O*-[6‴-*O*-acetyl-β-d-allopyranosyl-(1→2)]-β-d-glucopyranoside (ISC 1), and 4′-*O*-methylisoscutellarein-7-*O*-[6‴-*O*-acetyl-β-d-allopyranosyl-(1→2)]-β-d-glucopyranoside (ISC 2), since these were major compounds of the obtained extracts characterised by HPLC-DAD, and are known for significant biological activities—mainly anti-inflammatory. TP content was also recorded as one of the responses.

Experimental results of 30 runs for selected responses obtained under different experimental conditions are presented in Table 2.

Experimentally obtained values for output variables were fitted to a quadratic polynomial model. ANOVA was used to assess the effects of selected variables, interactions between variables, and the statistical significance of the model. Based on statistically significant *p*-values for the model (0.0075 for TP, 0.0002 for HYP, 0.0001 for ISC 1, and ˂ 0.0001 for ISC 2), it could be concluded that the quadratic polynomial model represented a good approximation for the investigated responses. This has been confirmed with reasonably high coefficients of multiple determinations (R^2^), whose values were 0.8162, 0.9064, 0.9118, and 0.9295 for TP, HYP, ISC 1, and ISC 2 content, respectively. A low value of coefficient of variation (CV < 10%) indicates good reproducibility of the responses [17]. CVs were slightly higher than 10%—10.80% for TP, 12.02% for HYP, 12.37% for ISC 1, and 12.79% for ISC 2—indicating an increase in the difference between the experimental and the obtained results. However, as the CVs were only slightly higher, these differences can be characterized as irrelevant errors. Lack of fit had insignificant *p*-values for TP (0.4323), HYP (0.1335), and ISC 2 (0.1734), which was also the evidence for the adequacy of the model. Lack of fit showed a significant *p*-value of 0.0316 for ISC 1. According to Mayers et al. [18], significant lack of fit indicates that the dispersion of the experimental design was a model-independent measure of the pure error.

*p*-values of regression coefficients for each of the investigated responses are summarized in Table 3.

### 2.1. Effect of Extraction Parameters on Total Phenolics Content

The content of total phenolics in *S. raeseri* extracts obtained using UAE varied between 19.32 and 47.23 mg GAE/g dw. These results were similar or higher compared with our results previously reported for wild-growing *S. raeseri* and *S. scardica* collected on the mountain Galičica, North Macedonia (18.25–20.28 mg GAE/g dw) [2]. Moreover, the obtained results are also comparable with those published in our previous paper, where we analysed samples of *S. raeseri subsp. raeseri* cultivated in Serbia (34.1 mg GAE/g in budding phase and 15.3 mg GAE/g in overflowering phase) [4]. Karapandzova et al. [19] studied TP content in seven wild-growing and one cultivated sample from North Macedonia, where a similar content was recorded, ranging from 47.57 to 50.87 mg GAE/g. On the other hand, Tunalier et al. [20] reported higher content of TP in *Sideritis* species that they analysed (i.e., *S. amasiaca, S. serratifolia, S. phlomoides*).

The highest TP content in this study was achieved using a 1:40 solid-to-solvent ratio (SSR), 70% ethanol, an extraction time of 50 min, and a temperature of 65 °C. On the other hand, the lowest content was obtained in an extract prepared using 10% ethanol, a 1:30 SSR, an extraction temperature of 50 °C, and a 35-min extraction time.

*p*-values of linear, interaction, and quadratic terms of regression coefficients for TP content are shown in Table 3. Linear and quadratic terms of ethanol concentration, as well as linear terms of extraction temperature and SSR, had a statistically significant influence on TP extraction, while linear and quadratic terms of extraction time, quadratic terms of extraction temperature and SSR, and interaction between factors did not have statistically significant influence. This means that the factors that have an impact on TP extraction are ethanol concentration, temperature, and SSR, while the extraction time, as well as interactions between each of the factors, have no impact. Based on the significant *p*-values, it can be concluded that the factors that affect the UAE of TP are the linear and quadratic terms of ethanol concentration (X_2_ and X_2_^2^), the linear terms of extraction temperature (X_4,_) and the linear terms of SSR (X_3_); thus, the mathematical equation describing the model for TP extraction has the form:

(1) $$TP=40.10+2X_{2}+3.91X_{3}+2.51X_{4}-2.80X_{2}{}^{2}$$

**Figure 1:**
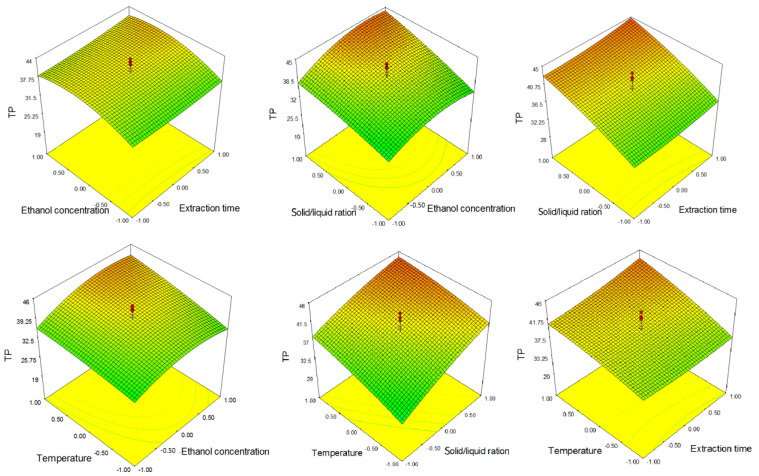
Response surfaces showing the combined effect of parameters on total phenolic content (TP).

The linear terms of extraction temperature and SSR positively affect the extraction of TP, which means that the increase in these parameters leads to the increase in the TP content in the obtained UAE extracts. The linear terms of ethanol concentration have a positive influence, while the quadratic terms have a negative influence on TP extraction; this indicates that an increase in ethanol concentration leads to increase in TP content to a certain point, but a further increase leads to a decrease in TP content. These observations can be seen in Figure 1.

From the response surfaces (Figure 1), it can be seen that an increase in ethanol concentration to approximately 80% (coded value 0.5) had an increasing effect on TP, and a further increase in ethanol concentration led to its slight decrease.

Tsibranska et al. [13] showed that TP and TF from cultivated hybrid *Sideritis scardica* × *Sideritis syriaca* were under the direct influence of ethanol concentration. They obtained maximum TP and TF amounts using a water–ethanol solvent ratio of 20:80. Moreover, in this study it was shown that 90% of the phenolics were extracted during the first 2.5 h. In our study, extraction time did not have significant influence on TP content.

The influence of SSR was also reported for other plant species. Živković et al. [21] pointed out SSR as one of the factors that positively influence the extraction of TP from pomegranate peel. Such results may be due to a greater amount of solvent that can penetrate into plant cells. UAE can contribute to the additional disruption of cell walls and better penetration of solvent into cells; thus, phenolic compounds can be released at higher levels from impaired cells [22].

In our samples, extraction temperature also had a positive influence on TP extraction. Tomšik et al. [23] showed that temperature has a significant effect on the mass transfer process, and can lead to a reduction in the viscosity of the solvent, as well as to the improvement of solvent penetration. Furthermore, degradation of cellular structures is higher at higher temperatures, which makes cells more permeable [24]. According to our results, we can conclude that the phenolics present in *S. raeseri* aerial parts are generally stable at high-temperature conditions. A similar trend of the influence of higher temperatures on the extraction of TP has been observed by other authors [21,25,26].

### 2.2. Effect of Extraction Parameters on Hypolaetin and Isoscutellarein Derivatives Content

The genus *Sideritis* is known as a rich source of biologically active flavonoids. Among dominant flavonoids in Central and Eastern Mediterranean *Sideritis* species, derivatives of hypolaetin (HYP) and isoscutellarein (ISC) were reported [1,2,4,13,27,28]. Güvenç et al. [29] showed that the hypolaetin 7-*O*-[6‴-*O*-acetyl-β-d-allopyranosyl-(1→2)-β-d-glucopyranoside] possesses high anti-inflammatory and antinociceptive activities compared to isoscutellarein derivatives. Moreover, antioxidant activity was also reported for HYP and ISC derivatives [13]. In our samples, HYP, ISC 1, and ISC 2 were dominant among flavonoids.

The content of HYP in *S. raeseri* extracts obtained using UAE varied from 1.05 to 11.46 mg/g dw. The obtained results are in accordance with our previous results obtained for *S. raeseri* and *S. scardica*, where total HYP content varied from 7.93 to 12.87 mg/g dw in 96% ethanolic extracts [2]. In our study, the highest content of HYP was achieved using a 1:40 SSR, 70% ethanol, an extraction time of 50 min, and a temperature of 65 °C. On the other hand, the lowest content was obtained in extract prepared using 10% ethanol, a 1:30 SSR, an extraction temperature of 50 °C, and a 35-min extraction time.

According to *p*-values for linear, interaction, and quadratic terms of regression coefficients present in Table 3, UAE of HYP from *S. raeseri*—taking into account only significant parameters—can be described by the equation:

(2) $$HYP=10.14+1.77X_{2}+0.57X_{3}+0.46X_{4}-1.27X_{2}{}^{2}$$

As in the case of TP, the linear terms of ethanol concentration, SSR, and temperature had a significant positive influence on HYP extraction, while the quadratic terms of ethanol concentration had a significant negative influence.

Extraction time, quadratic terms of extraction temperature and SSR, and interaction between factors had insignificant *p*-values, meaning that these parameters did not have any influence on the UAE of HYP.

#### -7-*O*- [6‴-*O*-acetyl-β-d-allopyranosyl (1 → 2)]-β-d-glucopyranoside (HYP)

**Figure 2:**
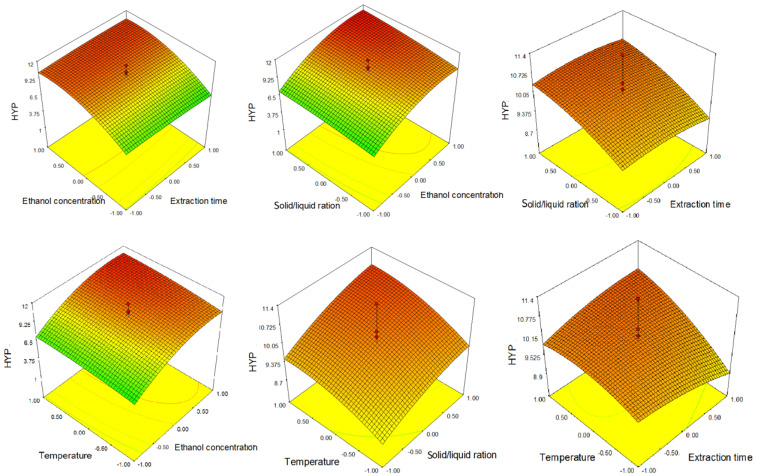
Response surfaces showing the combined effect of parameters on 4′-*O*-methylhypolaetin-7-*O*-[6‴-*O*-acetyl-β-d-allopyranosyl (1 → 2)]-β-d-glucopyranoside (HYP).

From the 3D graphs (Figure 2), the same trend of the influence of process parameters described by Equation (2) can be observed. Moreover, as noticed at TP response surfaces, an increase in ethanol concentration to approximately 80% had an increasing effect on phenolic content, and a further increase in ethanol concentration led to its slight decrease.

The content of ISC 1 in *S. raeseri* extracts obtained using UAE varied from 0.68 to 10.68 mg/g dw. The obtained results are in accordance with our previous results obtained for *S. raeseri* and *S. scardica*, where total ISC varied from 4.73 to 7.34 mg/g dw in 96% ethanolic extracts and 20 mg/g dw in water extract [2]. According to the results presented in Table 2, the highest content of ISC 1 was achieved using a 1:40 SSR, 70% ethanol, an extraction time of 20 min, and a temperature of 65 °C. On the other hand, the lowest content was obtained in an extract prepared using 10% ethanol, a 1:30 SSR, an extraction temperature of 50 °C, and a 35-min extraction time.

According to Table 3, the linear and quadratic terms of ethanol concentration and linear terms of extraction temperature had a significant influence. The quadratic terms of extraction temperature, linear and quadratic terms of extraction time and SSR, and interaction between factors did not have statistically significant influence on the UAE of ISC 1. The model describing the UAE of ISC 1 had the following form:

(3) $$ISC\;1=8.89+1.59X_{2}+0.79X_{4}-1.18X_{2}{}^{2}$$

**Figure 3:**
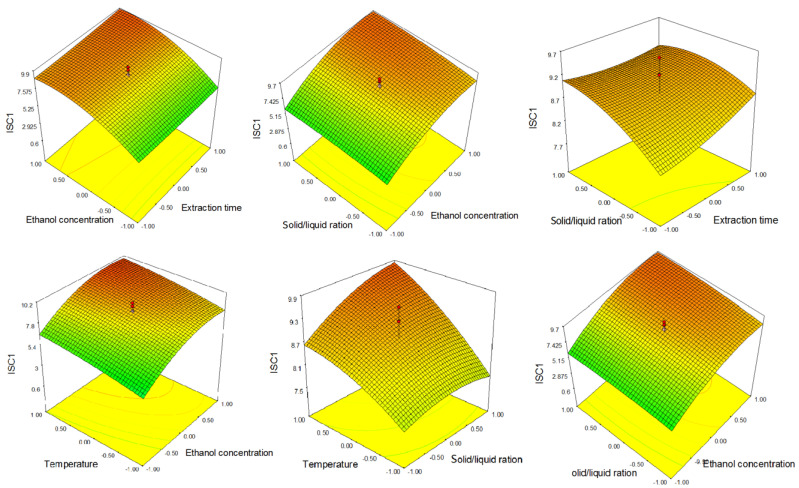
Response surfaces showing the combined effect of parameters on isoscutellarein 7-*O*-[6‴-*O*-acetyl-β-d-allopyranosyl-(1→2)]-β-d-glucopyranoside (ISC 1).

Ethanol concentration had the same influence on the ISC 1 extraction as on that of TP and HYP. The linear terms of ethanol concentration had a positive influence, while the quadratic terms had a negative influence, meaning that increase in ethanol concentration led to increase in the ISC 1 content in UAE to certain point, while further increase in ethanol concentration lead to a decrease in ISC 1 content. From Figure 3, it can be seen that ISC 1 content increases with increasing ethanol concentration to about 80%, after which a slight decrease can be observed. From Equation (3) and Figure 3, it can be seen that the temperature—as in the case of TP and HYP—had a positive influence on ISC 1 extraction.

The content of ISC 2 in *S. raeseri* extracts obtained using UAE varied from 0.74 to 15.56 mg/g dw. Similar to the case of ISC 1, these results are in accordance with our previous results obtained for *S. raeseri* and *S. scardica*, where total ISC varied from 4.73 to 7.34 mg/g dw in 96% ethanolic extracts and 20 mg/g dw in water extract [2]. The highest content of ISC 2 was achieved using a 1:40 SSR, 50% ethanol, an extraction time of 35 min, and a temperature of 50 °C. As in the case of other three compounds, the lowest content was obtained in the extract prepared using 10% ethanol, a 1:30 SSR, an extraction temperature of 50 °C, and a 35-min extraction time.

The extraction of ISC 2, taking into account the significance of all process parameters (Table 3), can be explained by the following equation:

(4) $$ISC\;2=12.74+2.81X_{2}+0.91X_{3}+X_{4}-1.69X_{2}{}^{2}$$

The terms that had a positive influence on the UAE of ISC 2 were the linear terms of ethanol concentration, linear terms of SSR, and linear terms of temperature, while the quadratic terms of ethanol concentration had a negative influence. Extraction time, interactions between each of the factors, and the quadratic terms of temperature and SSR did not have any influence on the extraction of ISC 2, due to their *p*-values being higher than 0.05. The same observations for the UAE parameters’ influence were noticed for TP, HYP, and ISC 2.

**Figure 4:**
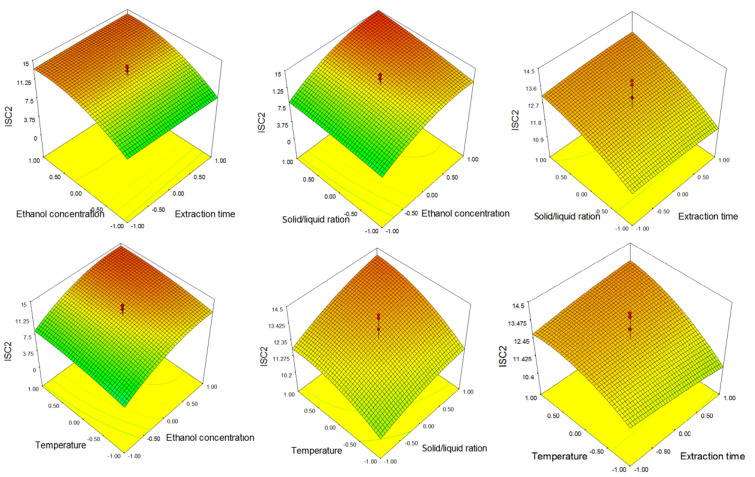
Response surfaces showing the combined effect of parameters on 4′-*O*-methylisoscutellarein-7-*O*-[6‴-*O*-acetyl-β-d-allopyranosyl-(1→2)]-β-d-glucopyranoside (ISC 2).

From the 3D graphs (Figure 4), the same trend of the influence of process parameters described by Equation (4) can be observed.

Ethanol concentration and extraction temperature had a statistically significant influence on the extraction of hypolaetin and isoscutellarein derivatives, while temperature had no statistically significant effect. SSR had a significant influence only on HYP and ISC1.

Temperature positively influenced the UAE of hypolaetin and isoscutellarein derivatives; a positive influence of temperature was also observed for TP. As previously mentioned, many authors reported a positive influence of temperature on the extraction process, as a result of a higher mass transfer process.

Ethanol concentration had a positive effect on the extraction of hypolaetin and isoscutellarein derivatives to a certain point (approximately 80%). Tsibranska et al. [13] reported that maximum flavonoid content was obtained when 80% ethanol was used as solvent. Hypolaetin and isoscutellarein derivatives belong to the group of flavonoids. The negative influence of ethanol concentrations higher than 80% could also be related to a reduction in ultrasonic cavitation due to reaching the boiling point of ethanol. The boiling point for 80% ethanol is in the range from 78 to 80 °C, which is the highest used temperature in this study.

As previously mentioned, a positive influence of SSR in UAE was reported by many authors [21,22].

### 2.3. Optimization of Extraction Parameters and Model Validation

After carrying out the experimental design for all of the investigated responses (TPC, HYP, ISC 1, and ISC 2 content), it was found that the optimal conditions for extraction of their maximal content from *S. raeseri* aerial parts are as follows: extraction time of 49.4 min, ethanol concentration of 65%, solid-to-solvent ratio of 1:39.5, and extraction temperature of 62.75 °C. The predicted values of investigated responses in the extract prepared at these conditions are 48.49 mg GAE/g dw for TP content, 11.98 mg/g dw for HYP content, 10.71 mg/g dw for ISC 1 content, and 16.11 mg/g dw for ISC 2 content.

Using the same extraction conditions, the predicted response values obtained by models under optimum conditions were checked experimentally. The results obtained during the validation of the optimized conditions were within the 95% confidence interval to predicted values, and amounted to 47.11 mg GAE/g dw, 11.73 mg/g dw, 9.54 mg/g dw, and 15.40 mg/g dw for TP, HYP, ISC 1, and ISC 2 content, respectively. This confirms that the selected RSM model was successfully applied for the UAE of *S. raeseri* aerial parts, in order to obtain extracts with maximal TPC and content of individual bioactive flavonoids—i.e., HYP, ISC 1, and ISC 2.

## 3. Materials and Methods

### 3.1. Plant Material, Standards, and Reagents

Aerial parts of *Sideritis raeseri* subsp. *raeseri* were collected in the phase of full flowering (July, 2019) on the natural locality of Sreden Vrv (altitude 1600–1660 m), Galičica mountain, North Macedonia. Voucher specimens Nos. S31/16–S33/16 were deposited at the Institute for Medicinal Plants Research, Belgrade, Serbia. Plant material was air-dried at room temperature for 4–6 days and ground using a laboratory mill. Following standards previously isolated and identified in our laboratories, the following were used: 4′-*O*-methylhypolaetin-7-*O*-[6‴-*O*-acetyl-β-d-allopyranosyl (1→2)]-β-d-glucopyranoside (HYP), isoscutellarein 7-*O*-[6‴-*O*-acetyl-β-d-allopyranosyl-(1→2)]-β-d-glucopyranoside (ISC 1), and 4′-*O*-methylisoscutellarein-7-*O*-[6‴-*O*-acetyl-β-d-allopyranosyl-(1→2)]-β-d-glucopyranoside (ISC 2) [2]. All reagents used for analytical procedures were of analytical grade: Folin–Ciocalteu phenol reagent, sodium carbonate, methanol, orthophosphoric acid, and formic acid were purchased from Sigma-Aldrich Chemie GmbH (Munich, Germany), while HPLC grade acetonitrile was purchased from Merck (Germany).

### 3.2. Experimental Design and Statistical Model

For the optimization of the UAE of dried aerial parts of *S. raeseri*, we used a central composite design (CCD); this design has the advantage of better prediction compared to some other designs [30], as well as its potential use in two-step sequential response surface methods. The design consisted of 30 randomized runs with 5 replicates at the central point. Four variables—i.e., extraction time, ethanol concentration, solid-to-solvent ratio, and extraction temperature—were selected as the responses in the designed experiment, where each of four variables was tested at five different levels. For statistical calculations, the variables were coded by the following equation:

X_i_ = (x_i_ − x_0_)/Δx_i_ (5)

where X_i_ is a coded value of the independent variable, x_i_ is the actual value of the independent variable, x_0_ is the actual value of the independent variable in the centre of the domain, and Δx_i_ is the step change value.

The model proposed for each response was:

Y = β_0_ + β_1_X_1_ + β_2_X_2_ +β_3_X_3_ + β_4_X_4_ + β_11_X_1_^2^ + β_22_X_2_^2^ + β_33_X_3_^2^ + β_44_X_4_^2^ + β_12_X_1_X_2_ + β_13_X_1_X_3_ + β_14_X_1_X_4_ + β_23_X_2_X_3_ + β_24_X_2_X_4_ (6)

where Y is the response; X_1_ is the extraction time; X is the ethanol concentration; X_3_ is the solid-to-solvent ratio; X_4_ is the extraction temperature; and β_o_ is the intercept, β_1_, β_2_, β_3_, and β_4_ are the linear, β_11_, β_22_, β_33_, and β_44_ the quadratic, and β_12_, β_13_, β_14_, β_23_, and β_24_ the interaction regression coefficient terms, respectively. Optimal extraction conditions were determined calculating the maximum content of TPC, HYP, ISC 1, and ISC 2 as responses. For the experimental design, data analysis, and determination of optimal conditions, Design-Expert V.7 Trial software (Stat-Ease, Minneapolis, MN, USA) was used. For the evaluation of the significance of independent variables’ influence and interactions, ANOVA was used (the differences were statistically significant at *p* < 0.05). The adequacy of the model was evaluated by the coefficient of determination (R^2^), and by *p*-values for the model and lack-of-fit testing.

The correctness of the model was verified by performing UAE at the defined optimal conditions (ethanol concentration, temperature, extraction time, and solid-to-solvent ratio) in order to obtain maximal TPC, HYP, ISC 1, and ISC 2 content. The experimentally obtained results and predicted values were compared and further analysed using Design-Expert V. 7 Trial.

### 3.3. Extraction

Different quantities of plant material (0.5–2.5 g) were mixed with 25 mL of solvent (ethanol 10–90%) using different solid-to-solvent ratios (SSRs)—i.e., 1:10–1:50. Samples were extracted in an ultrasonic bath (Bandelin, Sonorex) using various temperatures (from 20 to 80 °C) and time periods (from 5 to 65 min). Samples were filtered through filter paper after the extraction, and stored at −18 °C until further analysis.

### 3.4. Determination of Total Polyphenols

TP in *S. raeseri* extracts was determined spectrophotometrically using the Folin–Ciocalteu method [31]. For the calibration of a standard curve, gallic acid (0–100 mg/L) was used. The results were expressed as milligrams of gallic acid equivalents per gram of dry weight of sample (mg GAE/g dw). All experiments were repeated three times.

### 3.5. HPLC Analysis

Analyses were carried out on an Agilent 1200 RR HPLC instrument with a DAD detector (Agilent, Waldbronn, Germany) using a reversed-phase LiChrospher RP-18 (Agilent) analytical column (250 × 4 mm i.d.; 5 µm particle size). The mobile phase consisted of solvent A (1% *v/v* solution of orthophosphoric acid in water) and solvent B (acetonitrile). Separation was achieved according to the following scheme: 90–80% A 0–5 min, 80% A 5–10 min, 80–70% A 10–20 min, 70–30% A 20–30 min, 30–0% A 30–35 min. Detection wavelengths were set at 280 and 330 nm, and the flow rate was 1 mL/min. The injection volume was 5 μL, while the column temperature was maintained at 25 °C. The compounds 4′-*O*-methylhypolaetin-7-*O*-[6‴-*O*-acetyl-β-d-allopyranosyl (1→2)-β-d-glucopyranoside (HYP), isoscutellarein 7-*O*-[6‴-*O*-acetyl-ß-d-allopyranosyl (1→2)]-ß-d-glucopyranoside (ISC 1) and 4′-*O*-methylisoscutellarein-7-*O*-[6‴-*O*-acetyl-β-d-allopyranosyl-(1→2)]-β-d-glucopyranoside (ISC 2) were previously isolated in our laboratory [2]. The concentrations of compounds in the investigated extracts were determined using calibration curves (r > 0.9997). The results are presented as milligrams per gram of dry weight (mg/g dw).

## 4. Conclusions

Due to their long traditional use, complex chemical composition, and confirmed biological activities, *Sideritis* species attract increasing interest today. In our study, the effects of four parameters—i.e., extraction time, extraction temperature, ethanol concentration, and solid-to-solvent ratio—on the ultrasound-assisted extraction of *S. raeseri* aerial parts were evaluated using the response surface methodology. A multi-response optimization study based on a central composite design enables us to predict the optimal conditions for high-rate extraction of total phenols, 4′-*O*-methylhypolaetin-7-*O*-[6‴-*O*-acetyl-β-d-allopyranosyl (1→2)]-β-d-glucopyranoside, isoscutellarein 7-*O*-[6‴-*O*-acetyl-β-d-allopyranosyl-(1→2)]-β-d-glucopyranoside, and isoscutellarein 7-*O*-[6‴-*O*-acetyl-β-d-allopyranosyl (1→2)]-6′’’-*O*-acetyl-β-d-glucopyranoside—known as a contributors to the biological activities of *S. raeseri*. An extraction time of 49.40 min, an ethanol concentration of 65%, a solid-to-solvent ratio of 1:39.50, and an extraction temperature of 62.75 °C were proven to be optimal in the case of the *S. raeseri* phenolics extraction. *Sideritis* species are traditionally used as water extracts (teas), but according to our results, higher amounts of biologically active phenolics were extracted via less polar extracts. On the other hand, such extracts can be especially valuable to the pharmaceutical and cosmetic industries for the production of more complex preparations.

## Figures and Tables

**Table 1:** Coded and actual levels of independent variables for the designed experiment.

Input Variable	Symbol	Level				
		−2	−1	0	1	2
Extraction time (min)	X_1_	5	20	35	50	65
Ethanol concentration (%)	X_2_	10	30	50	70	90
Solid-to-solvent ratio (g/mL)	X_3_	1:10	1:20	1:30	1:40	1:50
Extraction temperature (°C)	X_4_	20	35	50	65	80

**Table 2:** Central composite design with UAE parameters and experimentally obtained values of TP, HYP, ISC 1, and ISC 2 ^a^.

Run	Extraction Time (min)	Ethanol Concentration [%]	Solid-to-Solvent Ratio (g/mL)	Temperature (°C)	TP (mg GAE/g dw)	HYP (mg/g dw)	ISC 1 (mg/g dw)	ISC 2 (mg/g dw)
1	5	50	01:30	50	39.14	10.88	9.54	14.42
2	20	30	01:20	35	34.49	6.75	6	7.02
3	20	30	01:20	65	31.48	5.68	5.06	6.22
4	20	30	01:40	35	36.1	6.2	4.2	5.56
5	20	30	01:40	65	42.39	7.89	7.93	9.58
6	20	70	01:20	35	30.43	8.86	6.94	10.41
7	20	70	01:20	65	34.29	8.24	8.06	10.91
8	20	70	01:40	35	40.83	8.97	7.34	11.46
9	20	70	01:40	65	40.77	10.48	10.68	14.2
10	35	10	01:30	50	19.32	1.05	0.68	0.74
11	35	50	01:10	50	28.87	8.74	7.71	10.93
12	35	50	01:30	20	29.29	9.05	7.53	10.41
13	35	50	01:30	50	42.24	11.34	9.2	13.9
14	35	50	01:30	50	39.87	10.18	9.56	13.03
15	35	50	01:30	50	36.27	9.65	8.66	11.96
16	35	50	01:30	50	37.26	9.58	8.55	11.71
17	35	50	01:30	50	41.69	9.68	8.16	12.17
18	35	50	01:30	50	43.25	10.39	9.2	13.69
19	35	50	01:30	80	45.31	10.37	9.07	13.02
20	35	50	01:50	50	44.43	11.12	8.78	14.43
21	35	90	01:30	50	36.98	10.2	8.17	13.29
22	50	30	01:20	35	32.37	5.09	4.18	4.61
23	50	30	01:20	65	35.64	7.14	7.42	8.25
24	50	30	01:40	35	38.92	6.42	5.05	6.49
25	50	30	01:40	65	37.83	6.84	6.78	8.65
26	50	70	01:20	35	25.48	7.33	7.8	9.19
27	50	70	01:20	65	40.06	10.61	10.03	13.59
28	50	70	01:40	35	42.78	10.32	8.74	13.43
29	50	70	01:40	65	47.23	11.46	10.09	15.56
30	65	50	01:30	50	43.32	9.36	9.58	12.55

^a^: TP: total phenolic content; HYP: 4′-*O*-methylhypolaetin-7-*O*-[6‴-*O*-acetyl-β-d-allopyranosyl (1→2)]-β-d-glucopyranoside; ISC 1: isoscutellarein 7-*O*-[6‴-*O*-acetyl-β-d-allopyranosyl-(1→2)]-β-d-glucopyranoside; ISC 2: 4′-*O*-methylisoscutellarein-7-*O*-[6‴-*O*-acetyl-β-d-allopyranosyl-(1→2)]-β-d-glucopyranoside.

**Table 3:** Corresponding *p*-values of linear, interaction, and quadratic terms of regression coefficients obtained for selected response variables (TP, HYP, ISC 1, and ISC 2) ^a^.

Term	Response			
	TP	HYP	ISC 1	ISC 2
**Linear**				
X_1_^b^	0.3807	0.8605	0.4147	0.9215
X_2_	0.0302	<0.0001	<0.0001	<0.0001
X_3_	0.0004	0.0193	0.1339	0.0065
X_4_	0.0092	0.0498	0.0014	0.0034
**Interaction**				
X_1_X_2_	0.5891	0.3316	0.3866	0.3613
X_1_X_3_	0.8162	0.8383	0.4669	0.6875
X_1_X_4_	0.3965	0.2194	0.7378	0.3045
X_2_X_3_	0.2345	0.4142	0.4848	0.2654
X_2_X_4_	0.3007	0.6001	0.9391	0.8925
X_3_X_4_	0.5819	0.7931	0.2578	0.5562
**Quadratic**				
X_1_^2^	0.5524	0.4861	0.5672	0.7984
X_2_^2^	0.0030	<0.0001	<0.0001	<0.0001
X_3_^2^	0.3950	0.3569	0.2446	0.3203
X_4_^2^	0.5156	0.2393	0.2737	0.0719

^a^: TP: total phenol content; HYP: 4′-*O*-methylhypolaetin-7-*O*-[6‴-*O*-acetyl-β-d-allopyranosyl (1→2)]-β-d-glucopyranoside; ISC 1: isoscutellarein 7-*O*-[6‴-*O*-acetyl-β-d-allopyranosyl-(1→2)]-β-d-glucopyranoside; ISC 2: 4′-*O*-methylisoscutellarein-7-*O*-[6‴-*O*-acetyl-β-d-allopyranosyl-(1→2)]-β-d-glucopyranoside. ^b^: X_1_: extraction time; X_2_: ethanol concentration; X_3_: solid-to-solvent ratio; X_4_: extraction temperature.

## Data Availability

The data used to support the findings of this study are included within the article.

## Sample Availability

Samples of the compounds are not available from the authors.
